# Prediction of measles elimination in Côte d’Ivoire by 2030

**DOI:** 10.4102/jphia.v17i1.1703

**Published:** 2026-05-22

**Authors:** Kouadio D. Ekra, Calixte H.H. Guehi, Eric M. Ahoussou, Guillaume Okoubo

**Affiliations:** 1 Department of Public Health, Faculty of Medical Sciences, Université Félix Houphouët-Boigny, Abidjan, Côte d’Ivoire; 2 National Institute of Public Hygiene, Abidjan, Côte d’Ivoire

**Keywords:** measles, Côte d’Ivoire, prediction, incidence, 2025–2030

## Abstract

**Background:**

The WHO African Region Strategic Plan for Immunisation 2021–2030 aims to eliminate measles in at least 80% of countries by 2030. What could the situation in Côte d’Ivoire be like?

**Aim:**

To predict the incidence of measles by 2030.

**Setting:**

Côte d’Ivoire, a country in West Africa.

**Methods:**

National routine measles surveillance data were analysed using seasonal time series models. Stationarity was assessed using standard unit root tests, and seasonality was addressed through appropriate differentiation. Structural breaks were examined using break and stability tests, which identified a major break around 2020. An Autoregressive Integrated Moving Average (ARIMA) model incorporating an intervention variable was adjusted to account for this change. Model performance was evaluated using cross-validation and compared to a naive seasonal benchmark. Projections were generated through 2030. The annual incidence per 1 000 000 population was estimated.

**Results:**

A significant structural break around 2020 was identified. Projections show a slight downward trend in incidence for the overall population. However, among children, an increase in incidence from 146 to 164 by 2030 was observed. During cross-validation, the ARIMA model with intervention did not outperform the naive seasonal benchmark for short-term forecasts.

**Conclusion:**

By 2030, Côte d’Ivoire will not have eliminated measles, with a high incidence among children. Sustained high vaccination coverage, strengthened surveillance, and scenario-based planning remain critical to achieving measles elimination.

**Contribution:**

The study guides decision-makers on the prospects and the necessity of adopting innovative strategies aimed at eliminating measles.

## Introduction

The discovery of the measles vaccine by Enders and its approval in 1963, followed by its widespread use through national vaccination programmes since 1974, has been a public health success story.^1^ The measles vaccine had reduced morbidity and mortality by 78% and 88%, respectively, worldwide by 1995, compared to the pre-vaccination period.^2^ Countries in the Americas, such as the United States, Canada and Cuba, have undertaken strategic vaccination policies (catch-up campaigns for children aged 9 months to 14 years, regardless of their previous vaccination status or history of measles) that have significantly reduced the transmission of the disease. Indeed, in 1994, the countries of the WHO American region set the goal of eliminating measles by the year 2000, a regional aim that was achieved in 2002,^3^ thanks to the application of cutting-edge strategies that have made it possible to almost completely stop the transmission of measles.

The elimination of measles is defined as the interruption of measles transmission in a defined geographical area for at least 12 months and is considered verified if this interruption is maintained for a period of at least 36 months.^4^ This interruption of transmission must be combined with a high-quality surveillance system, supported by laboratories and with sufficient sensitivity and specificity to detect, notify and investigate suspected cases and outbreaks in a timely manner.^5^

Member states across all WHO regions have adopted measles elimination goals to be achieved by 2020 or earlier, with the following specific objectives: (1) to reduce the incidence of measles in all countries; (2) to increase access to immunisation services in all districts; (3) to improve coverage during all scheduled measles supplementary and outbreak response immunisation activities; and (4) to improve the quality of measles surveillance, as well as epidemiological and virological investigations of measles outbreaks in all countries. The achievement of elimination was verified in the Region of the Americas in September 2016.^6^ On the other hand, in countries in the WHO African region that have also adopted and subscribed to the elimination of measles, the regional incidence was 38.4 cases per 1 million inhabitants in 2020,^7^ in view of which the elimination target was set again for 2030.^8,9^

In Côte d’Ivoire, after a decline in the incidence of measles between 2013 and 2015, we are seeing a resurgence of the disease with an incidence of more than 10 cases per 1 million inhabitants. Sadly, investigations remain weak in our regions, making it difficult to combat this disease.^10,11^

This study aimed to analyse the evolutionary trends of measles from 2010 to 2024 in Côte d’Ivoire with a view to producing a 6-year projection.

### Objectives

#### General objective

To predict the incidence of measles over the next 6 years using the Autoregressive Integrated Moving Average (ARIMA) model.

#### Specific objectives

The specific objectives were:

- To identify the appropriate ARIMA prediction model.
- To predict the incidence of measles over the next 6 years in the general population.
- To predict the incidence of measles over the next 6 years in children aged 0–15 years.

## Research methods and design

### Study design

A predictive study was conducted based on an analysis of all clinically and laboratory-confirmed measles cases reported by health districts in Côte d’Ivoire between 2010 and 2024.

### Study setting

Côte d’Ivoire is located in western sub-Saharan Africa and covers an area of 322 462 km^2^. It is bordered by Burkina Faso and Mali to the north, Liberia and Guinea to the west, Ghana to the east and the Gulf of Guinea to the south. The country has four seasons, namely a long rainy season from March to June, a short dry season from July to August, a short rainy season from September to October and a long dry season from November to February. The total population living in Côte d’Ivoire is estimated to be 29 389 150 inhabitants, with a density of 91 inhabitants/km^2^ and an average annual growth rate of 2.9%.^10^ The health system comprises 33 health regions and 113 health districts.

### Data sources

Measles surveillance data in Côte d’Ivoire from 2010 to 2024 were used to adjust the ARIMA model. To record these data, health centres report suspected measles cases to health districts, which in turn notify the central body (Directorate for the Coordination of the Expanded Program on Immunisation [DC-PEV]). Laboratory confirmation of the diagnosis is made by the Pasteur Institute of Côte d’Ivoire (IPCI) based on the detection of immunoglobulin M (IgM). All these data are stored in the Epi.info database of the DC-PEV and are validated through harmonisation sessions with different structures of the health system (the DC-PEV, the National Institute of Public Hygiene [INHP]), the IPCI and the Directorate of Health Information (DIS):

- **Observation period:** The daily measles case notification data were reported for the period 2010 to 2024.
- **Collected variables:** Date of notification, suspected measles case, final classification of measles diagnosis, age, vaccination status and district of notification.

### Data exploration

Raw data on measles surveillance in Côte d’Ivoire are collected daily by health districts. These data were aggregated on a half-yearly basis by grouping them by year and half-year and summing up the number of measles cases. This choice was made in order to smooth out daily fluctuations and, above all, the seasonal dynamics of measles in Côte d’Ivoire. The time series used for model adjustment is therefore semi-annual. The time series used for model adjustment is therefore semi-annual.

### Stationarity test

An exploratory analysis of the data was performed by visualising the trend and seasonality with graphs (time series curves, Autocorrelation Function [ACF] and Partial Autocorrelation Function [PACF]) and checking the stationarity of the series with the Dickey–Fuller test^11^ after transforming the data into a time series using the ‘ts()’ function in R software.

This time series of measles cases was decomposed using the R function ‘decompose’, making it possible to identify the trend and seasonality. The number of differentiations was automatically calculated using the ndiffs and nsdiffs functions for non-seasonal and seasonal differentiations, respectively. It was subsequently applied two differentiations to remove the trend and retested the stationarity on the differentiated series using the Dickey–Fuller test (significance threshold 5%).

### Model identification

It was adjusted for seasonality (since seasonal cycles were present) by automatically identifying the best^12,13^ ARIMA model. Model stationarity was checked using model residuals, and the best-fitting model was automatically identified using the auto.arima function.

As the model identified by auto.arima does not take double differentiation into account, we tested a set of models that do take double differentiation into account. The model with the lowest Root Mean Squared Error (RMSE) and whose Akaike Information Criterion (AIC) and residuals were white noise was selected.

### Model validation and evaluation

The model was validated using a cross-validation approach adapted to time series, in particular, rolling-origin cross-validation, which was used to assess the model’s predictive performance. This method evaluates the stability of the model in different temporal contexts, such as the coronavirus disease 2019 (COVID-19) pandemic period.

The model’s performance was evaluated using RMSE, Mean Absolute Error (MAE) and Theil U test compared to a naive seasonal reference (snaive).

Calculating accuracy metrics between the training data and the validation data allowed us to evaluate the accuracy of the model based on the smaller values with the validation data test than the training data test (RMSE, MAE and Theil U test). The Theil inequality coefficient is most often used for model validation by evaluating the accuracy of the model’s prediction. It is a coefficient whose values range from 0, meaning a perfect prediction, to 1, representing maximum inequality.^14^

### Sensitivity analysis and structural breaks

It was conducted to assess the robustness of the model in the face of potential structural breaks in the time series. This involved re-estimating the model over different periods, with a particular focus on the period of the COVID-19 pandemic.

### Future forecasts

A seasonal ARIMA model was estimated over the entire series by including a dummy variable coded 0 before rupture and 1 after rupture. This variable was introduced as an external regression (xreg) to represent the level change.

Based on the selected and validated ARIMA model, we made half-yearly predictions on the number of measles cases from 2025–2030 in Côte d’Ivoire, taking into account the uncertainty of the forecasts (confidence intervals). Taking into account the population data, from the general population census of Côte d’Ivoire, and especially on the projections of this population (2025–2030) transmitted by ANSAT to the Ministry of Health, both for the general population and for children aged 0–15 years.^10^ For these years (2025–2030), we calculated the annual incidence of measles. These predictions were made for the general population and for children aged 0–15 years.

All analyses were performed using R.4.2.1 software, including the prediction of the number of measles cases using the R ‘forecast’ function.^15^

### Ethical considerations

This study used routinely collected aggregate surveillance data that did not contain any personally identifiable information. As such, the analysis posed minimal risk to individuals. Data were processed in accordance with national regulations and institutional guidelines on data protection and confidentiality. Given the use of anonymised secondary data, no formal informed consent was required. Ethical approval to conduct this study was obtained from the Research Ethics Committee of the National Institute of Public Health. The ethical clearance number is 001-2025.

## Results

From 2010–2024 a total of 41 251 suspected cases of measles were reported in Côte d’Ivoire, with 9738 (24%) confirmed cases. Of the latter, 8795 (90%) were children aged 0 to 15 years.

### Decomposition of the time series of measles cases

**FIGURE 1:**
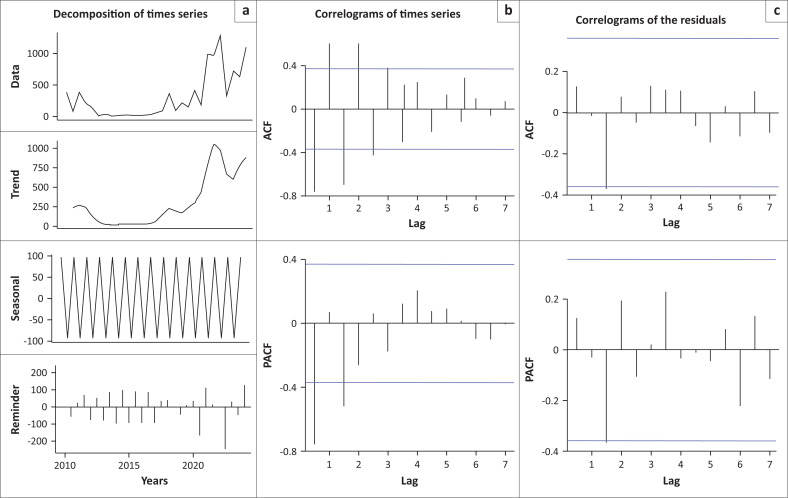
(a) Decomposition of the time series of measles cases; (b) Correlograms of the time series of measles cases; (c) Correlograms of the residuals obtained after series adjustment, complete data, Côte d’Ivoire 2010–2024.

The decomposition of the time series generated three main components, namely an ascending trend from 2017, seasonality with periodic fluctuations on a biannual basis and high residuals in 2020, 2021 and 2022, suggesting unusual events not explained by the other components. These findings are the same as the complete data (Figure 1a) and those of children aged 0–15 years (Online Appendix 1 Figure OA1-2a).

Lags 1 and 2 show significant positive autocorrelations, with the decrease in autocorrelations after a few lags indicating that the series may be stationary (Figure 1b and Figure 4b).

Automatic identification of the number of differentiations noted one non-seasonal differentiation (*d* = 1) and one seasonal differentiation (*d* = 1).

It should be noted that the series required two differentiations to be stationary (test of Dickey-Fuller, *p* = 0.03).

The ACF and PACF of the residuals show that most of the autocorrelations are not significant (Figure 1c).

Some residual correlations at specific lags might suggest incomplete adjustment; however, the Ljung–Box test indicates that the residuals do not exhibit significant correlation (*p* = 0.13).

### Choice of models

It initially used the auto.arima function to select the model. However, the model proposed by auto.arima() (ARIMA(0,0,1)(1,1,0)[2]) did not take into account the two differentiations. We then compared five models based on double differentiation and low AR and MA orders (between 0 and 2) to avoid overfitting. (Online Appendix 1 Table OA1-1).

The ARIMA(1,1,1)(0,1,1)[2] model was chosen based on its smaller RMSE.

After adjusting the model over the entire series, there is a significant ACF peak (Online Appendix 1 Figure OA1-1). We then decided to increase q by +1 in order to capture this residual dependence.

Finally, it was applied this ARIMA(1,1,2)(0,1,1)[2] model to the entire data series.

The selected SARIMA model (ARIMA(1,1,2)(0,1,1)[2]) is good, considering the following observations:

Indeed, the model residuals do not show significant autocorrelation as indicated by the Ljung-Box test (*p* = 0.05698 (> 0.05)). The residuals are therefore similar to white noise, which indicates that the model captures the main structures of the data well.

The chosen model has a simple structure: ARIMA(1,1,2)(0,1,1)[2] where (1,1,2) indicates a first-order moving average in non-seasonal data with a non-seasonal difference of order 1 and (0,1,1)[2] indicates a first-order seasonal autoregressive component with a seasonal difference of order 1 and a semi-annual seasonality ([2]).

### Model accuracy assessment

**FIGURE 2:**
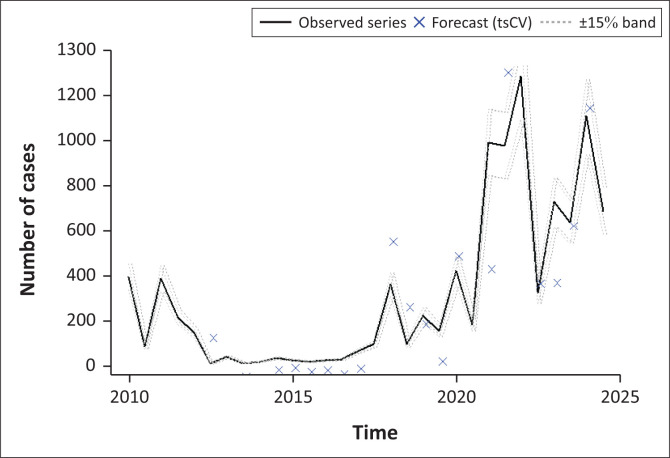
Comparison of observed and predicted measles cases, general population, Côte d’Ivoire 2023–2024.

Both predicted and observed data diverge with seasonal variations. Moreover, observed data remain within the confidence interval of predicted data (Figure 2).

For the training data, the RMSE and MAPE are respectively 215.8% and 107.6%. Similarly, the results for the test data are respectively 169.2% and 22.2%, and the proposed model performs better than the basic one.

Figure 2 compares observed values with forecasts validated by cross-checking at one step. Most forecasts fall within a tolerance band of ±15% during stable periods (before 2018), while deviations increase during periods of high volatility, particularly from 2020 onwards, indicating reduced accuracy for sudden peaks.

Furthermore, the higher RMSE (approximately 290 cases) compared to the MAE (185 cases) reveals the presence of significant errors visible during the sharp peaks after 2020, highlighting the model’s difficulty in anticipating outbreaks.

### Sensitivity analysis and structural breaks

It was therefore checked for structural breaks using the strucchange() package before performing an ARIMAX analysis.

This check noted that the second half of 2020 was the main structural break with the smallest Bayesian Information Criterion (BIC) value (413.1) (Online Appendix 1 Table OA1-2).

### Model fit quality with integrated break

The inclusion of a break indicator variable in the ARIMA model (ARIMA(1,1,2)(0,1,1)[2] + break dummy) made it possible to capture a significant structural change that occurred in the second half of 2020. This coefficient associated with the intervention (xreg = 607.7) indicates an increase in the average number of measles cases per half-year after that date. Furthermore, the inclusion of this break improved the model’s performance (RMSE fell from 290 to 170, a reduction of more than 40%). Furthermore, the Ljung-Box test had a *p*-value of 0.2055; the residuals are therefore white noise. This confirms the relevance of the model with intervention in this time series of measles data.

However, in cross-validation, the naive seasonal benchmark outperformed the ARIMA model with intervention (Theil’s *U* = 1.28) (Online Appendix 1 Table OA1-3), indicating superior short-term predictive accuracy. Nevertheless, the ARIMA model was selected for inference and scenario analysis because it explicitly accounts for the structural break identified in 2020 and provides interpretable estimates of post-break level changes.

### Forecasting measles cases with the fitted Autoregressive Integrated Moving Average model

**FIGURE 3:**
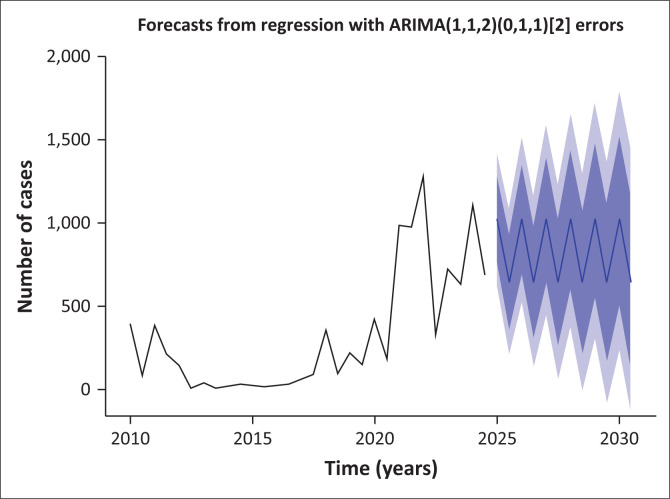
Predicted biannual measles cases in the general population in Côte d’Ivoire 2025–2030.

After 2025, the model predicts periodic fluctuation around a stable mean for the number of cases. The confidence interval increases over time, reflecting increasing uncertainty in predictions as the time horizon extends (Figure 3).

The final fitted model was used to generate forecasts for the period 2025–2030.

It is noted that the predicted incidence of measles will remain high in the general population, even if a downward trend will be observed. Indeed, the incidence will vary from 51.5 to 45.6 cases per million from 2025 to 2030 in the Ivory Coast (Table 1).

**TABLE 1:** Prediction of measles cases and incidence from 2025 to 2030 in the general population in Côte d’Ivoire.

Year	Population	Predicted cases per half-year	Predicted cases per year	95% CI	Annual incidence*	95% CI
2025	32 494 020	1026	1675	1085–2264	51.5	33.4–69.7
		649	-	-	-	-
2026	33 293 101	1022	1670	958–2382	50.2	28.8–71.6
		648	-	-	-	-
2027	34 090 660	1021	1668	852–2483	48.9	25.0–72.8
		647	-	-	-	-
2028	34 874 612	1020	1666	751–2581	47.8	21.5–74.0
		646	-	-	-	-
2029	35 679 380	1019	1664	651–2676	46.6	18.3–75.0
		645	-	-	-	-
2030	36 475 852	1018	1662	554–2770	45.6	15.2–75.9
		644	-	-	-	-

CI, confidence interval.

* , Number of cases per million.

In children aged 0–15, the incidence will be higher in 2025 by around 146 cases per million and will increase by 18 points to 160 cases per million in 2030 (Table 2).

**TABLE 2:** Prediction of measles cases and incidence from 2025 to 2030 in children aged 0 to 15 years in Côte d’Ivoire.

Year	Population	Predicted cases per half-year	Predicted cases per year	95% CI	Annual incidence*	95% CI
2025	12 256 825	1153	1790	1195–2385	146	97.5–195
		637	-	-	-	-
2026	12 552 703	1148	1828	1002–2654	146	79.8–211
		680	-	-	-	-
2027	12 868 694	1203	1928	926–2930	150	71.9–228
		725	-	-	-	-
2028	13 074 926	1257	2027	836–3219	155	63.9–246
		771	-	-	-	-
2029	13 317 564	1310	2127	734–3520	160	55.1–264
		817	-	-	-	-
2030	13 614 853	1362	2226	621–3831	164	45.6–281
		865	-	-	-	-

CI, confidence interval.

* , Number of cases per million.

## Discussion

This study provides a time series-based assessment of measles trends in Côte d’Ivoire and offers projections for the elimination of measles by 2030. By combining seasonal ARIMA modelling with explicit consideration of a structural break, the analysis highlights both the temporal dynamics of measles transmission and the considerable uncertainty surrounding future trajectories.

One of the main findings of this study is the identification of a structural break around 2020, corresponding to a marked change in the level and variability of reported measles cases. This break is plausible from an epidemiological standpoint and likely reflects a combination of factors, including disruptions to routine vaccination services, changes in healthcare-seeking behaviour, and surveillance sensitivity during the COVID-19 period. Incorporating this break using an intervention variable significantly improved the model fit, although this model performs less well than the naive seasonal model.

Our study population is mainly made up of children aged 0–15 years whose numerical weight has certainly influenced the type of ARIMA(1,1,3)(0,1,1)[2]+ break dummy model identified, and therefore the general trends of predicted measles cases in the general population and particularly in the age group itself. Thus, the predictions show that the number of cases is systematically higher in the first half of the year, suggesting a higher rate of transmission at the beginning of the year. The number of predicted cases for the second half of the year is significantly lower than that for the first half, reflecting a seasonal variation in measles transmission.

Some studies, such as one conducted in Cameroon, a country in sub-Saharan Africa, have noted this same significant trend in measles cases during the first half of each year, followed by a lower number of cases in the second half. This seasonality of measles was not only related to the annual rainfall pattern but also to the movement of young children with their mothers during the annual agricultural cycles^16^; we also noted mass movements of populations during the end-of-year holidays and religious festivals. Among the factors in the spread of measles, we can cite the school calendar, which has been identified by analysis factors underlying seasonal trends in England. This study noted an increase in transmission three times a year, coinciding with the start of school terms, and decreases with school holidays,^17^ which means that reducing contact between children during school holidays lowers the transmission of human-to-human infectious diseases such as measles and the varicella-zoster virus.^18^

The ARIMA model is generally used for short-term forecasting. The actual data did not match the model’s predicted data perfectly, but they were within the expected confidence interval. However, these predicted data give us an idea of how the incidence of measles will change over the next 6 years in Côte d’Ivoire. The predicted annual incidence in the general population remains high even though we observe its gradual decrease over the years. Indeed, the incidence will decrease from 51.5 cases per million in 2025 to 45.6 cases per million in 2030. This gradual decline in incidence is more related to the gradual increase in population than to a marked decrease in measles cases; the number of cases has remained virtually constant over the years. However, the predictions show a higher vulnerability of children aged 0–15 years. The incidence among children (146 cases per million in 2025) is significantly higher than in the general population (51.5 cases per million in 2025). This confirms that children are the group most at risk of measles, which is probably linked to insufficient vaccination coverage,^19^ late administration of vaccine doses or a rise in the number of susceptible children.

It should be noted that a population’s susceptibility to measles depends on measles vaccination (dose 1 and dose 2), vaccine efficacy, immunity acquired following previous infection, and protection conferred by maternal antibodies in infants.^20^ Furthermore, unlike the general population, the incidence among children will increase despite the growth of their population over the years. The results indicate that measles will continue to affect children significantly in Côte d’Ivoire up until 2030. Current efforts, while effective in reducing the overall incidence, are insufficient to eliminate the disease. Indeed, with a predicted incidence of 51.87 cases per million in 2025 and a one-point decline in the annual incidence observed with the analysis, Côte d’Ivoire will not achieve elimination by 2030. At this rate, and without significant changes in measles prevention strategies, it will take approximately 50 years to eliminate measles in Côte d’Ivoire.

To achieve the goal of eliminating measles in Côte d’Ivoire, it will be necessary to develop innovative strategies, in addition to investing in measles vaccination and surveillance programmes to significantly reduce the susceptible population. This involves extending measles vaccination campaigns, which were previously limited to children aged 0 months to 59 months, to children up to the age of 15 years. In light of these findings, it is important to include measles elimination in the National Health Development Plan (NHDP) for optimal programming of funding for innovative strategies.

### Study limitations

This study has certain limitations that must be taken into account when interpreting projections for the elimination of measles in Côte d’Ivoire by 2030.

Firstly, the analysis is based on systematically collected national surveillance data, which may be affected by under-reporting and differences in case detection between health regions and over time.

Secondly, the chronological approach assumes that historical trends, seasonality and the identified structural break will persist in the future. However, changes in the performance of vaccination programmes, the implementation of additional vaccination activities, population mobility or the capacity to respond to epidemics could significantly alter the future dynamics of measles.

Thirdly, the models do not incorporate certain key variables such as national vaccination coverage, disruptions to the health system, or behavioural factors influencing vaccine uptake. It should also be noted that the 95% confidence intervals for incidence remain wide, reflecting considerable uncertainty about future transmission patterns. Therefore, the results should be interpreted as scenario-based projections to support strategic planning and programme decision-making, rather than definitive predictions about measles elimination. Continued strengthening of surveillance systems and routine immunisation remains essential to validate and update these projections.

### Recommendations and strategies

To reduce the impact of measles and achieve its elimination in the general population, and specifically in children aged 0 to 15 years, three levels of intervention can be considered, namely increasing vaccination coverage, strengthening surveillance and conducting certain studies; in particular, studies evaluating current vaccination strategies and the age of measles vaccination in Côte d’Ivoire.

Increasing measles vaccination coverage should be effective through the implementation of routine vaccination that guarantees the administration of both doses of measles vaccine and targeted campaigns, reaching rural areas and vulnerable populations. This requires controlling the denominator through well-conducted target enumeration. Strengthening the immunity of the most at-risk age group is an imperative that requires the organisation of a multi-age measles vaccination campaign that includes children aged from 9 months to 15 years.

Also, for these different activities, including awareness raising, critical periods (before and during the first semester) should be targeted to take into account seasonal variations. Back-to-school periods should also be considered in the implementation of activities.

Disease surveillance – particularly for measles – will need to be strengthened to improve reporting in all health areas and on a continuous basis through strengthening health infrastructure, staff capacity and educating parents.

Finally, studies should be carried out to elucidate the socio-demographic and immunological profile of people aged over 15 years who have measles in order to take targeted action against them.

Understanding and acting on the determinants of vaccine hesitancy to improve Vaccine Coverage (VC), and understanding the perception of the importance of measles among stakeholders in order to improve the quality of surveillance.

## Conclusion

Data show that measles will remain a major challenge in Côte d’Ivoire up until 2030, with a relatively high incidence. The differences between semesters and the persistence of cases indicate the need for innovative vaccination and surveillance measures. Targeted, seasonal action focused on at-risk children could significantly reduce the impact of measles in this critical age group.

Continued monitoring and adjustments in measles control strategies will be essential for reducing its impact in the coming years.

Autoregressive Integrated Moving Average models applied to historical measles surveillance data are an important tool for predicting the occurrence of this disease.
